# Gramicidin S and melittin: potential anti-viral therapeutic peptides to treat SARS-CoV-2 infection

**DOI:** 10.1038/s41598-022-07341-x

**Published:** 2022-03-02

**Authors:** Mohammed Ghalib Enayathullah, Yash Parekh, Sarena Banu, Sushma Ram, Ramakrishnan Nagaraj, Bokara Kiran Kumar, Mohammed M. Idris

**Affiliations:** CSIR-Centre for Cellular and Molecular Biology, Hyderabad, Telangana 500007 India

**Keywords:** Cell biology, Drug discovery

## Abstract

The COVID19 pandemic has led to multipronged approaches for treatment of the disease. Since de novo discovery of drugs is time consuming, repurposing of molecules is now considered as one of the alternative strategies to treat COVID19. Antibacterial peptides are being recognized as attractive candidates for repurposing to treat viral infections. In this study, we describe the anti-SARS-CoV-2 activity of the well-studied antibacterial peptides gramicidin S and melittin obtained from *Bacillus brevis* and bee venom respectively. The EC_50_ values for gramicidin S and melittin were 1.571 µg and 0.656 µg respectively based on in vitro antiviral assay. Significant decrease in the viral load as compared to the untreated group with no/very less cytotoxicity was observed. Both the peptides treated to the SARS-CoV-2 infected Vero cells showed viral clearance from 12 h onwards with a maximal viral clearance after 24 h post infection. Proteomics analysis indicated that more than 250 proteins were differentially regulated in the gramicidin S and melittin treated SARS-CoV-2 infected Vero cells against control SARS-CoV-2 infected Vero cells after 24 and 48 h post infection. The identified proteins were found to be associated in the metabolic and mRNA processing of the Vero cells post-treatment and infection. Both these peptides could be attractive candidates for repurposing to treat SARS-CoV-2 infection.

## Introduction

The pandemic caused by SARS-CoV-2 has led to intense research not only on the biology of the virus but also therapeutic interventions with a multi-pronged approach^1^. Vaccines have been developed at “warp” speed. The overall efficacies of different vaccines, though variable are excellent^2^ and have played a major role in controlling the disease^3^. However, vaccines are not available universally and there have been cases of infection with SARS-CoV-2 even in vaccinated individuals, though not severe^4^. Also, the effect of vaccines would wane over time. The vaccines may also be less effective against newly emerging strains such as omicron which has a very large number of substitutions in the viral genome as compared to the earlier strains (https://www.cdc.gov/coronavirus/2019-ncov/science/science-briefs/scientific-brief-omicron-variant.html). There is clearly a need for development of therapeutic agents in addition to vaccines. In the area of anti-infective agents against SARS-CoV-2, efforts have been taken to generate therapeutic antibodies that would neutralize the virus and prevent its interaction with cellular receptors to gain entry into cells^5^. Considering the time scales in developing a drug de novo, there have been several attempts to re-purpose drugs to treat COVID19^1,6,7^. However, repurposed drugs have had very limited success in treating SARS-CoV-2 infection including remdesivir^8^. There is no drug to-date that can be used specifically to treat COVID19 disease. Although two drugs from Pfizer (Paxlovid) and Merck (Molnupiravir) appear to show promise, their effectiveness is still to be established unequivocally^9^.

Infection in the case of SARS-CoV-2 is initiated by binding of the spike protein to ACE2 followed by a series of steps leading to fusion and internalization of the virus and propagation^1,10^. If the binding of the spike protein to ACE2 is prevented, then the virus will no longer be able to enter cells and propagate. SARS-CoV-2 is an enveloped virus where the RNA is encapsulated within a lipid vesicular structure with the spike protein decorating on the external side giving the “corona” appearance^1,10^. Disruption of the lipid structure would lead to the disintegration of the virus. Naturally occurring membrane-active peptides have potent antimicrobial activity which stems from their ability to disrupt bacterial membranes^11,12^. We have explored the antiviral activity of two extensively studied peptides, gramicidin S having the sequence: [cyclo-(Val-Orn-Leu-D-Phe-Pro)2]^13^ and the bee venom peptide, melittin having the sequence: GIGAVLKVLTTGLPALISWIKRKRQQ-amide^14^. We reasoned that if the peptides could destabilize the viral membrane, the virus would disintegrate and would thus be rendered inactive. The peptides could also conceivably bind to the spike protein and prevents its interaction with ACE2 or inhibit fusion. Peptides are crucial components of host-defense against bacteria and fungi in species across the evolutionary scale^11,12^. There has been intense research in recent years to examine whether these and related peptides have the ability to neutralize SARS-CoV-2 and also have therapeutic potential^12,15–17^. They include naturally occurring peptides that can be easily isolated. Many of these peptides have also been investigated structurally. These include gramicidin S and the bee venom peptide melittin^13,14^. The antiviral activity of melittin against several viruses has been investigated extensively^18^. Nano-conjugates of melittin with sitagliptin have been investigated for anti-SARS-CoV-2 activity^19^. Other pharmacological activities of melittin have also been investigated^20,21^. Gramicidin S has been used therapeutically to treat dental applications in humans^22^.

The antiviral activity of melittin has been reported previously. Hood et al*.*, reported that melittin is highly effective in reducing HIV-1 infectivity^23^. Uddin et al., reported antiviral effect of melittin on different viruses (VSV-GFP, HSV-GFP, EV71, H3-GFP, RSV-GFP) both in vitro and in vivo^24^. Several studies showed melittin is effective against diverse array of viruses such as coxsackievirus, enterovirus, influenza A viruses, human immunodeficiency virus (HIV), herpes simplex virus (HSV), Junín virus (JV), respiratory syncytial virus (RSV), vesicular stomatitis virus (VSV), and tobacco mosaic virus (TMV)^18^. Not much scientific evidences or reports are available so far for the antiviral activity of the gramicidin S. Gramicidin S showed cell cytotoxicity (CC50) at 18.7 μg/ml in HT-29 cells^25^. Melittin showed CC50 of 6.45 μg/ml in C654 cells^26^.

We have investigated the antiviral activity of gramicidin S and melittin against SARS-CoV-2 in vitro in detail. We have observed that both the peptides have the ability to neutralize the virus in an in vitro assay using Vero cells. At the EC_50_ value, there is no cytolytic activity. The viral load had decreased drastically in the treatment group as seen by confocal microscopic images. Proteomic analysis indicates that there is also a metabolic effect and not merely viral lysis. Both the peptides could be attractive candidates for development as therapeutic agents to treat SARS-CoV-2 infection. As the viral membrane would be a likely target, mutant strains may also be susceptible to the peptides.

## Results

### Antiviral activity of peptides

**Figure 1:**
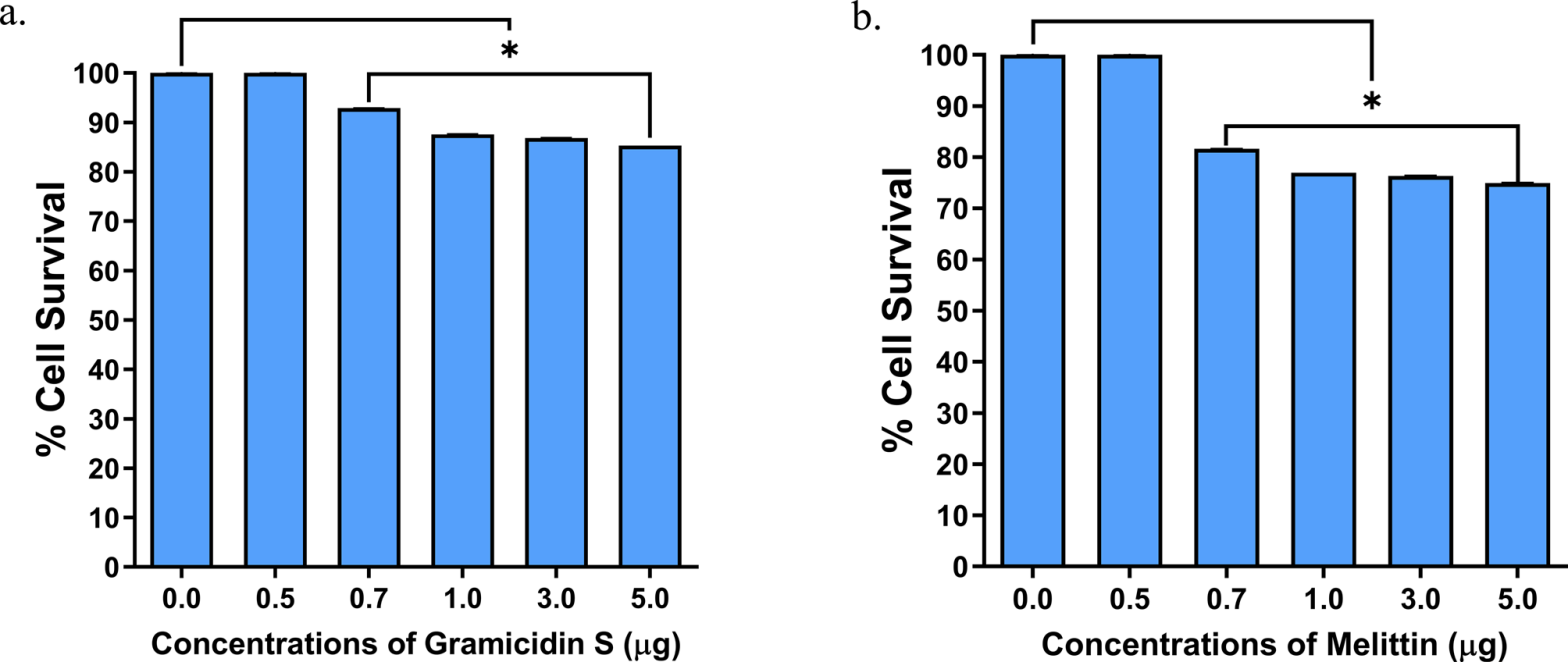
Measurement of Cytolytic activity of gramicidin S and melittin using MTT Assay: The graphs represent the percentage of cell viability vs. concentrations of (**a**) Gramicidin S (µg). (**b**) Melittin (µg). The values represent Mean ± SD of atleast three independent experiments. The data analysis and graphs were generated using GraphPad Prism (*Ver* 8.4.2). Significance of variance, p < 0.05 is considered statistically significant.

**Figure 2:**
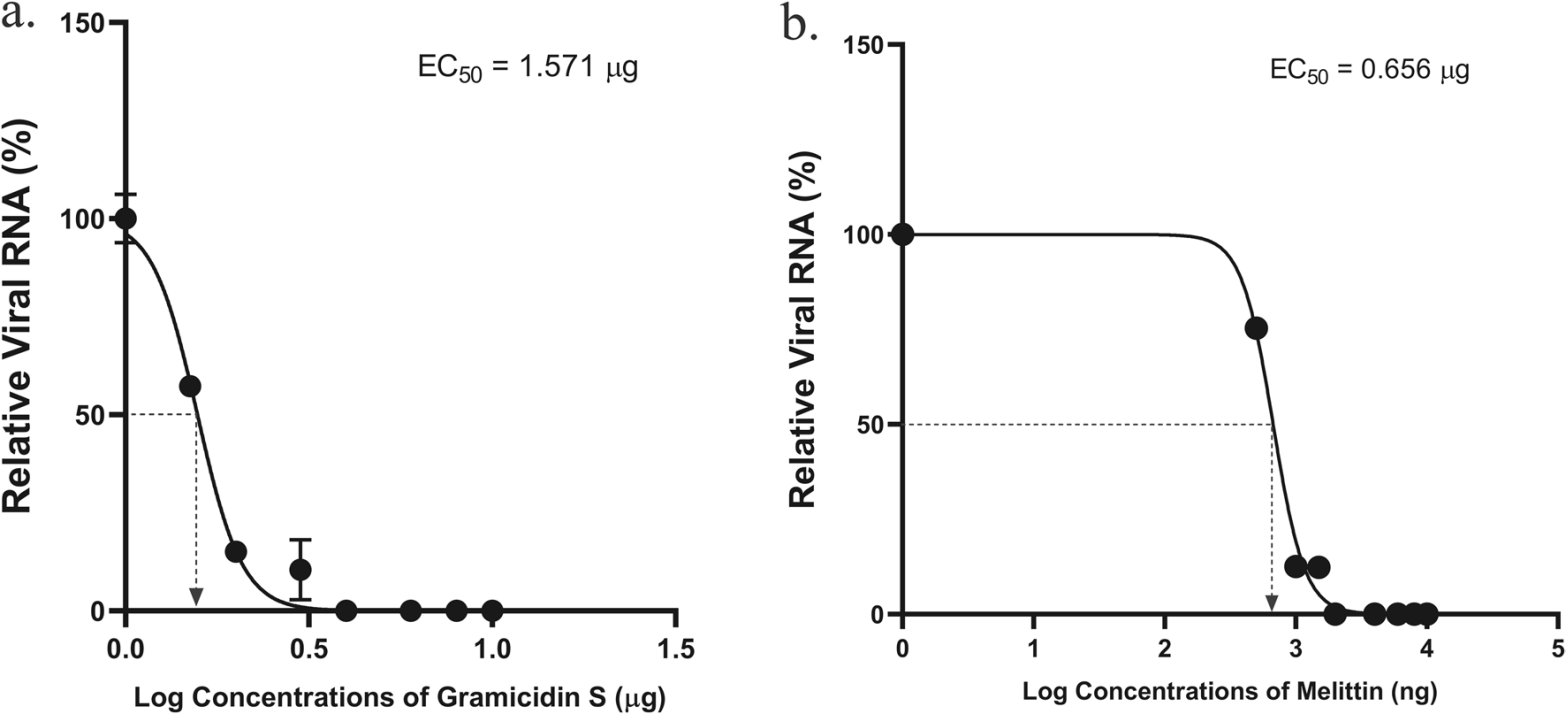
Anti SARS-CoV-2 activity of gramicidin S and melittin in vitro: (**a**). Relative viral RNA (%) vs. Log concentrations of gramicidin S (µg). (**b**) Relative viral RNA (%) vs. Log concentrations of melittin (ng). The graphs represent the Ct values of N-gene calculated using RT-qPCR in the supernatants. The Log EC_50_ value of gramicidin S (0.1963) corresponds to 1.571 µg and Log EC_50_ value (2.826) of melittin corresponds to 0.656 µg as shown in the graph.

**Figure 3:**
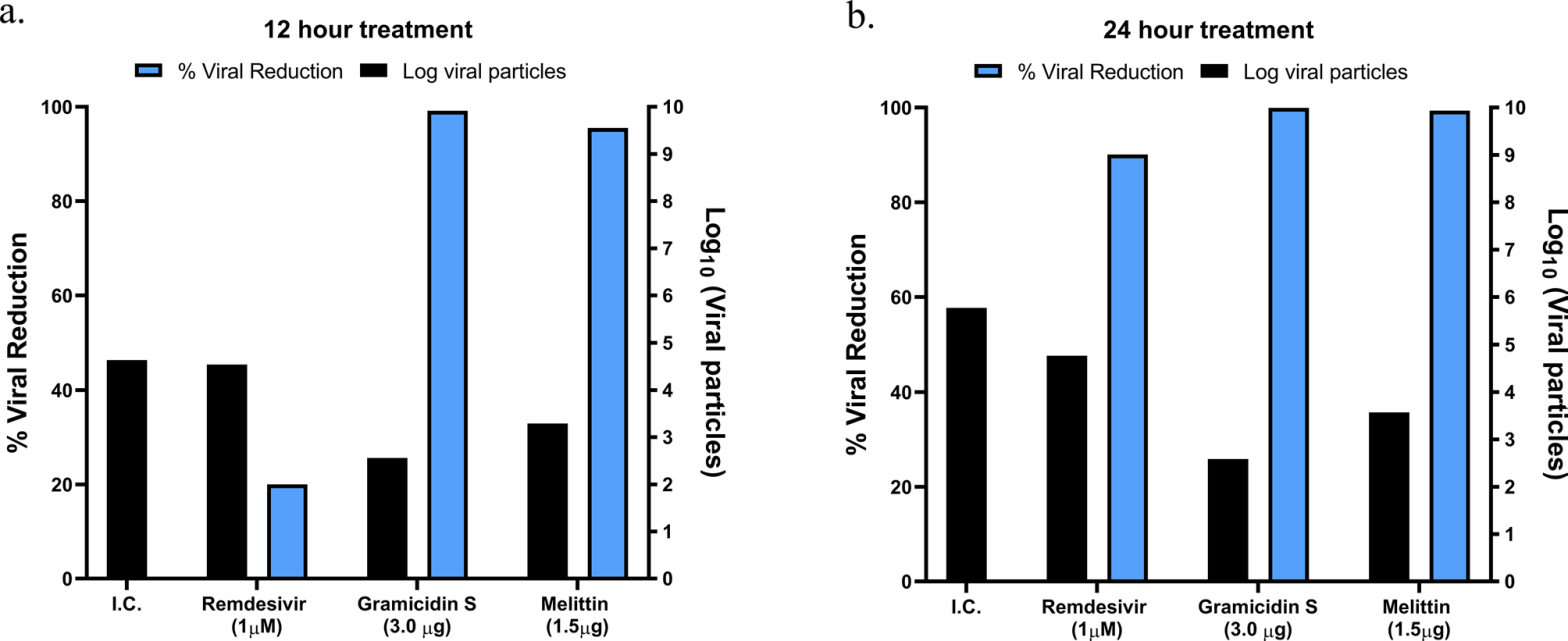
The anti SARS-CoV2 activity of remdesivir gramicidin S and melittin at 12 (**a**) and 24 (**b**) h post infection. The X axis represents different experimental groups: I.C (infection control), remdesivir (1 µM), gramicidin S (3.0 µg), and melittin (1.5 µg). The Y axis (Blue bars) represents % of viral reduction and Y’-Axis (black bars) represents the Log_10_ viral particles.

**Figure 4:**
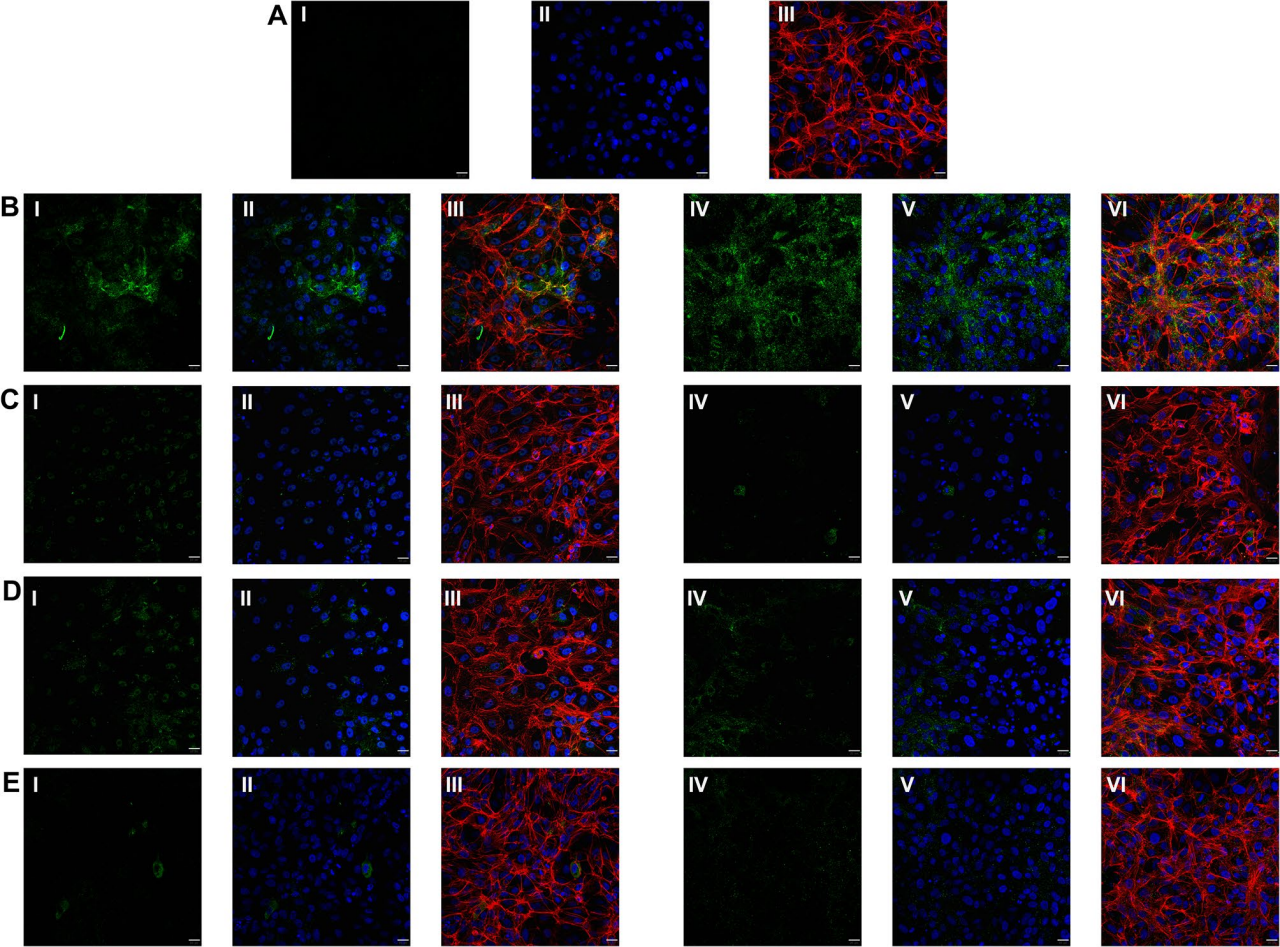
Immunofluorescence staining images against RBD protein expression specific to SARS-CoV-2 in Vero cells. (**A**) (I) Vero cells without SARS-CoV-2 infection (mock), (II) DAPI staining representing nucleus (blue); (III) Merged image of f-Actin (Phalloidin staining, red), nucleus (DAPI, blue). (**B**) Vero cells infected with SARS-CoV-2, (I) represents RBD protein expression (green) of SARS-CoV-2 virus at 12 h; (II) DAPI staining representing nucleus (blue) along with RBD protein expression of SARS-CoV-2 (green) at 12 h; (III) Merged image of f-Actin (red) (Phalloidin staining), nucleus (DAPI) and RBD protein of SARS-CoV-2 (green) at 12 h; (IV) represents RBD protein expression (green) of SARS-CoV-2 virus at 24 h; (V) DAPI staining representing nucleus (blue) along with RBD protein expression of SARS-CoV-2 (green) at 24 h; (VI) Merged image of f-Actin (Phalloidin staining, red), nucleus (DAPI, blue) and RBD protein of SARS-CoV-2 (green) at 24 h. (**C**) Vero cells infected with SARS-CoV-2 treated with gramicidin S, (I) RBD protein expression (green) of SARS-CoV-2 virus at 12 h; (II) DAPI staining representing nucleus (blue) along with RBD protein expression of SARS-CoV-2 (green) at 12 h; (III) Merged image of f-Actin (red) (Phalloidin staining), nucleus (DAPI) and RBD protein of SARS-CoV-2 (green) at 12 h; (IV) represents RBD protein expression (green) of SARS-CoV-2 virus at 24 h; (V) DAPI staining representing nucleus (blue) along with RBD protein expression of SARS-CoV-2 (green) at 24 h; (VI) Merged image f-Actin (Phalloidin staining, red), nucleus (DAPI, blue) and RBD protein of SARS-CoV-2 (green) at 24 h. (**D**) Vero cells infected with SARS-CoV-2 treated with melittin. (I) represents RBD protein expression (green) of SARS-CoV-2 virus at 12 h; (II) DAPI staining representing nucleus (blue) along with RBD protein expression of SARS-CoV-2 (green) at 12 h; (III) Merged image of f-Actin (red) (Phalloidin staining), nucleus (DAPI) and RBD protein of SARS-CoV-2 (green) at 12 h; (IV) represents RBD protein expression (green) of SARS-CoV-2 virus at 24 h; (V) DAPI staining representing nucleus (blue) along with RBD protein expression of SARS-CoV-2 (green) at 24 h; (VI) Merged image of f-Actin (Phalloidin staining, red), nucleus (DAPI, blue) and RBD protein of SARS-CoV-2 (green) at 24 h. (**E**) Vero cells infected with SARS-CoV-2 treated with remdesivir, (I) represents RBD protein expression (green) of SARS-CoV-2 virus at 12 h; (II) DAPI staining representing nucleus (blue) along with RBD protein expression of SARS-CoV-2 (green) at 12 h; (III) Merged image of f-Actin (red) (Phalloidin staining), nucleus (DAPI) and RBD protein of SARS-CoV-2 (green) at 12 h; (IV) represents RBD protein expression (green) of SARS-CoV-2 virus at 24 h; (V) DAPI staining representing nucleus (blue) along with RBD protein expression of SARS-CoV-2 (green)at 24 h; (VI) Merged image of f-Actin (Phalloidin staining, red), nucleus (DAPI, blue) and RBD protein of SARS-CoV-2 (green) at 24 h. Scale bars, 20 µm (40 × image).

The cytolytic activity of gramicidin S and melittin was determined using MTT assay (Fig. 1). Results showed 75–80% cell survival at all the concentrations tested (up to 5 µg) indicating the safe use of these peptides (Fig. 1). The SARS-CoV-2 viral particles enumerated by the RT-qPCR showed that treatment of gramicidin S and melittin effectively reduced viral load in vitro Log EC_50_ value of gramicidin S (0.1963) corresponds to 1.571 µg and Log EC_50_ value (2.826) of melittin corresponds to 0.656 µg (Fig. 2) The antiviral activity of gramicidin S (3.0 µg) and melittin (1.5 µg) at 12 and 24 h was examined along with remdesivir (1 µM) as assay control. The data shown in Fig. 3 indicates that the peptides show antiviral activity at 12 h and is more pronounced at 24 h. The gramicidin S and melittin showed 99% and 95% viral reduction respectively at 12 h compared with remdesivir (20%). At 24 h remdesivir showed 90% viral reduction whereas both gramicidin S and melittin showed 99% viral reduction. The SARS-CoV-2 antiviral activity of gramicidin S and melittin was compared with remdesivir by confocal microscopy (Fig. 4). Panel B (green fluorescence) indicates infection of cells, more prominent at 24 h. Panels D and E correspond to virus incubated with gramicidin S and melittin before incubating with cells. The considerable decrease in green fluorescence indicates that both the peptides have good anti-viral activity as with remdesivir shown in Panel E. Although, antiviral activity is observed at 12 h, it is more pronounced at 24 h.

### Proteomic analysis

The effect of the peptides on the ability of virus to infect Vero cells was studied by high-throughput proteomic analysis. iTRAQ based quantitative proteomics analysis identified 7 SARS-CoV-2 proteins as up regulated in the control infected Vero Cells. Nsp9, ORF1ab, ORF10 and nucleocapsid phosphoprotein were found to be up-regulated in the Vero cells after 24 h of infection, whereas the same proteins were found to be down regulated in the cells upon gramicidin S and melittin treatment (Table 1). Similarly, at 48 h post infection (hpi), ORF1ab, S protein, nucleocapsid phosphoprotein, helicase and RNA-dependent RNA polymerase were found to be up-regulated in the infected Vero cells which were found to be down regulated in the gramicidin S and melittin treated Vero cells (Table 1).

**Table 1:**
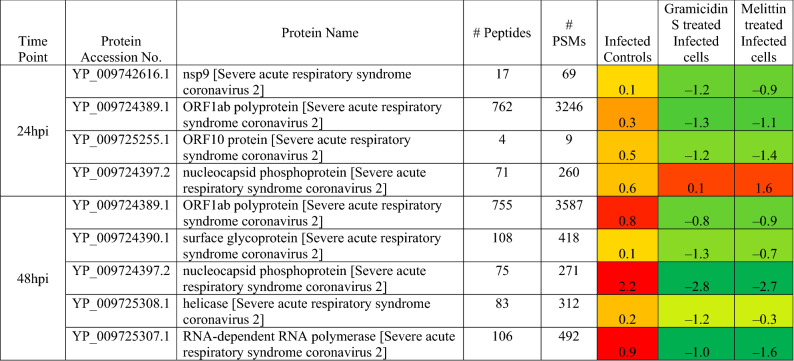
List of SARS-CoV-2 proteins and their expression level in the vero cells post infection (control), gramicidin S treatment and melittin treatment.

**Figure 5:**
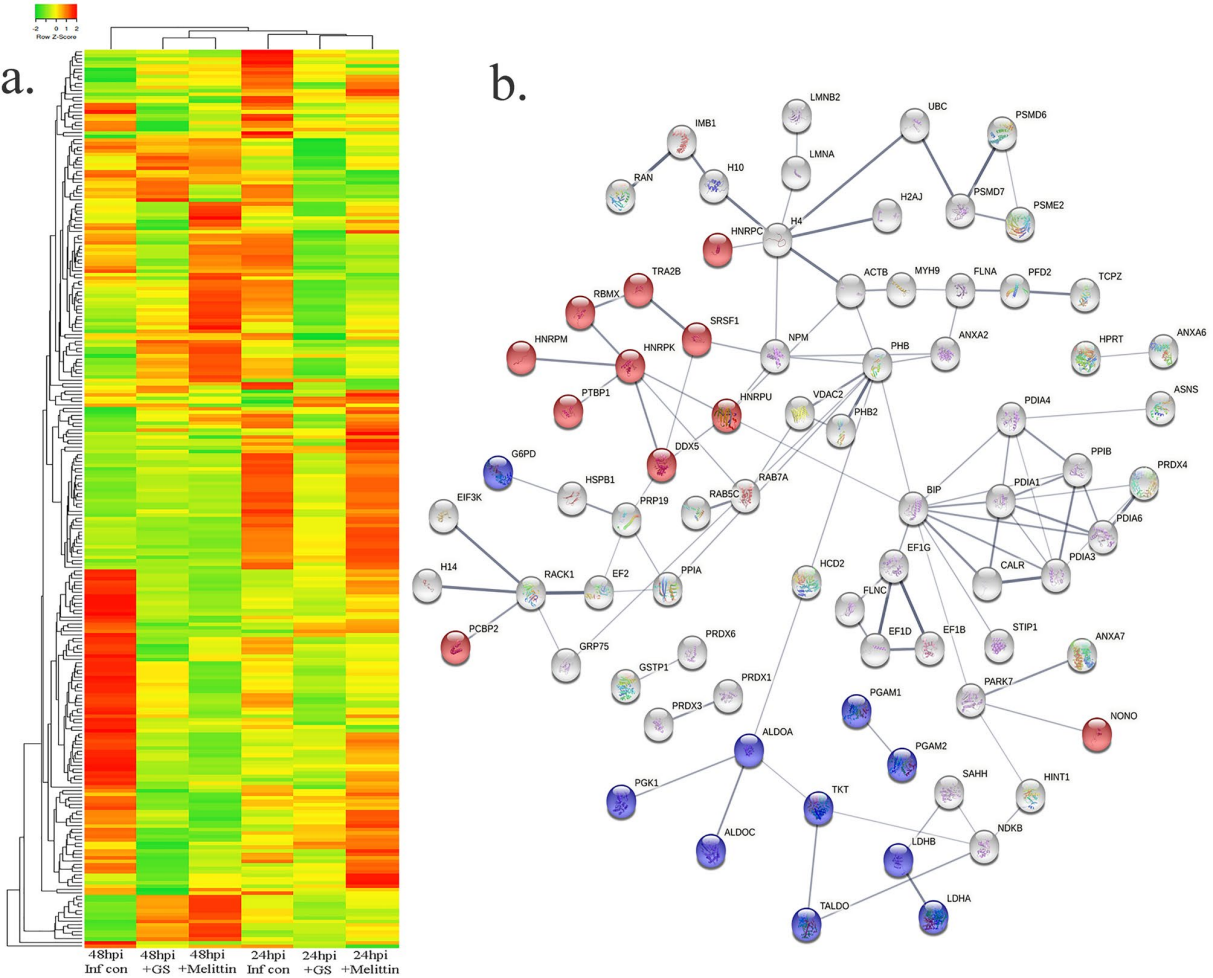
(**a**) Heat map expression of proteins in Vero cells post infection and peptide treatment. The image was created using web-enabled heatmapper software. (**b**) Network pathway analysis of the differentially expressed protein based on STRING analysis. The image was created using web-enabled STRING v11 software.

Based on proteomics analysis, a total of 254 proteins were found to be differentially regulated and associated in infected and peptide treated Vero cells (Table 2 and Fig. 5a). It was found that majority of up and down regulated proteins were reversed with gramicidin S and melittin treatment at 24 and 48 hpi. RS28, K22E, K2C1, RL17 are few of the major down-regulated proteins which were found to be reversing their expression post peptide treatment. NPM, ACLY, CALX and F184B were found to be up-regulated in Vero cells after 24hpi, whereas peptide treatment showed reversal of the protein expression (Table 2). Heat map analysis showed that gramicidin S and melittin-treated cells at their respective time point post infection are rooted together against out rooting with infected cells for the same time point post infection. It is very interesting to see the tight association of peptide treated Vero cell protein expression against control infected Vero cell protein expression for both the time points post infection (Fig. 5a).

**Table 2:**
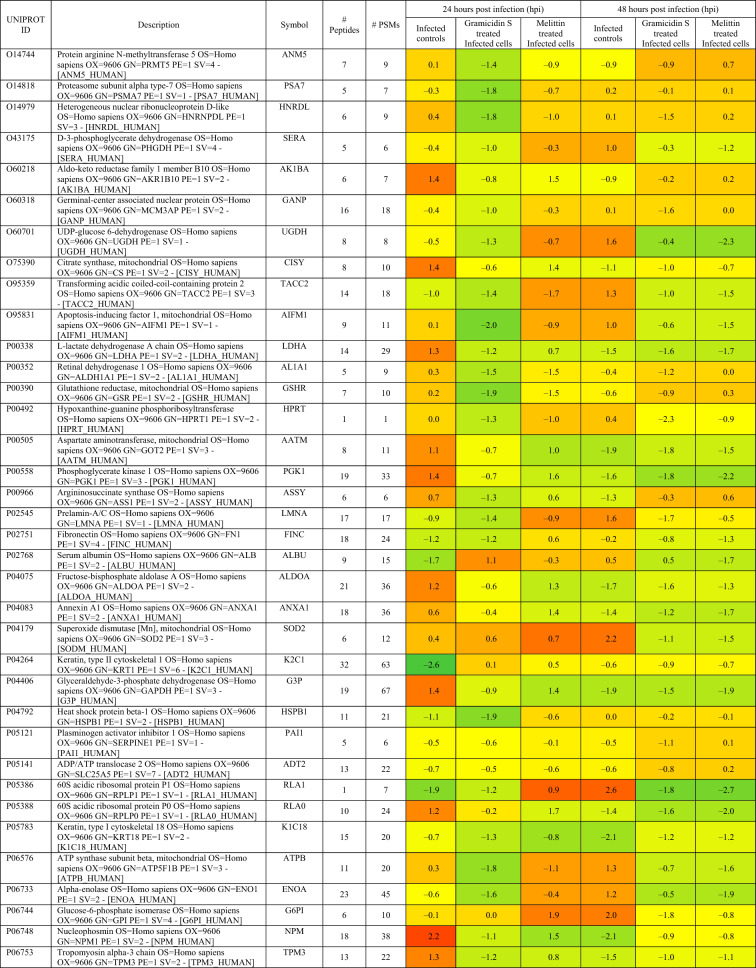

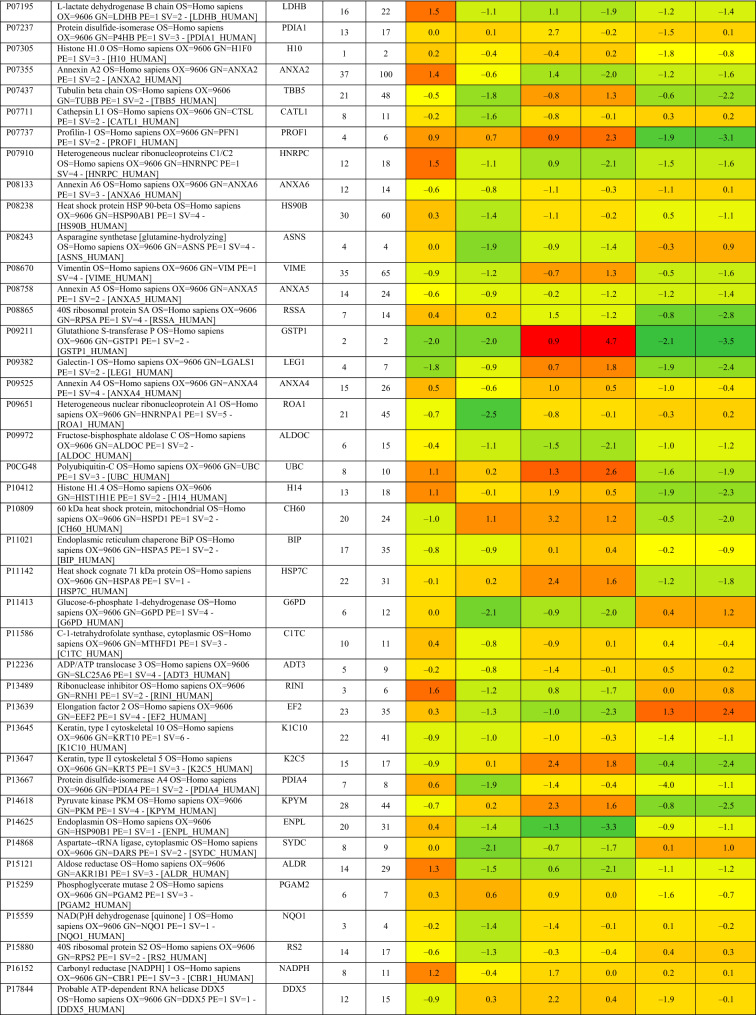

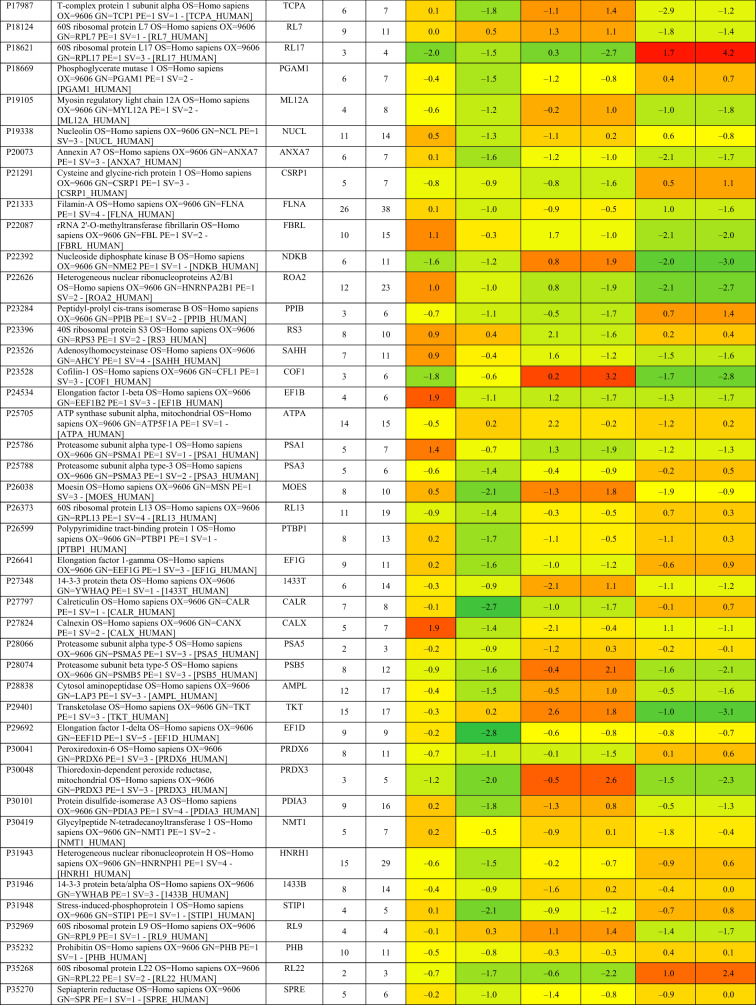

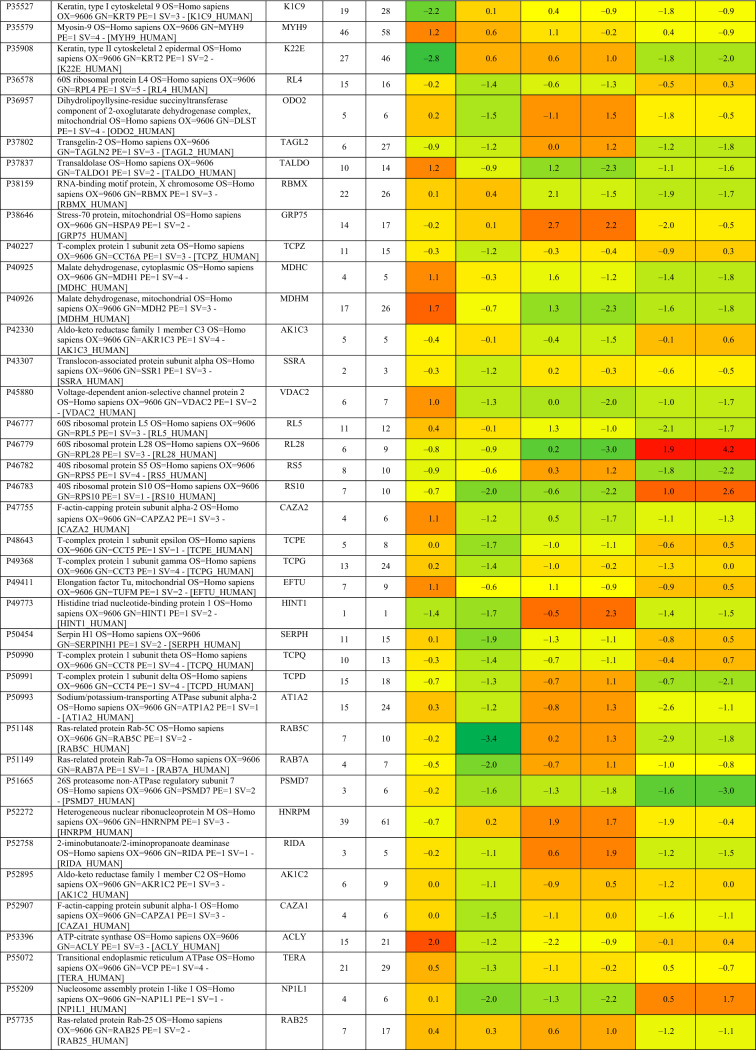

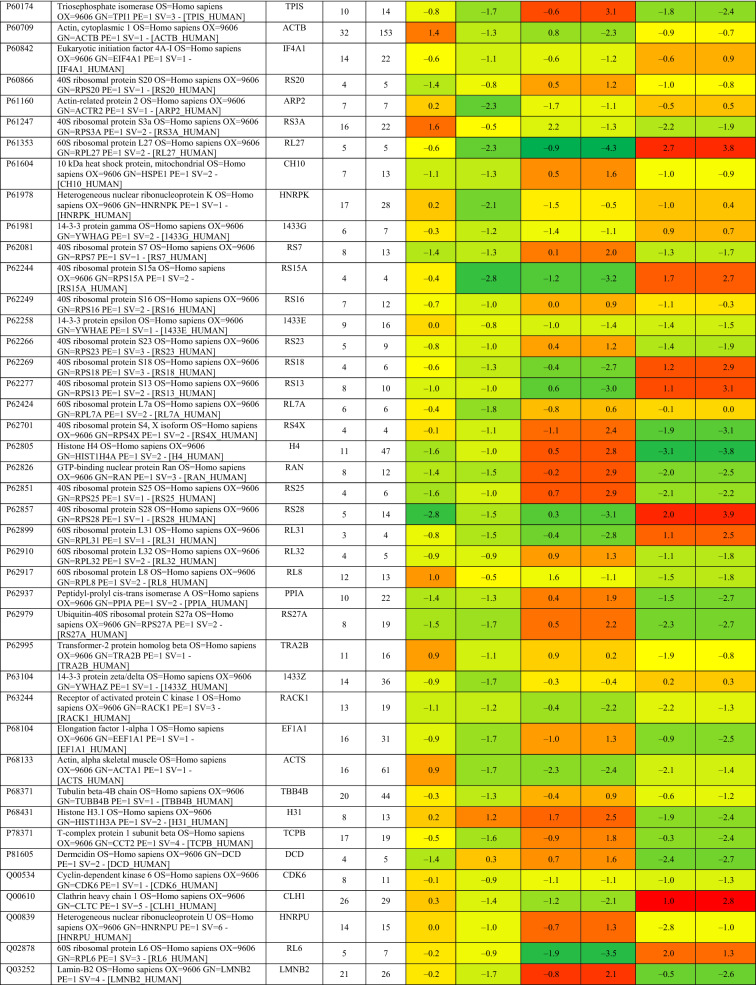

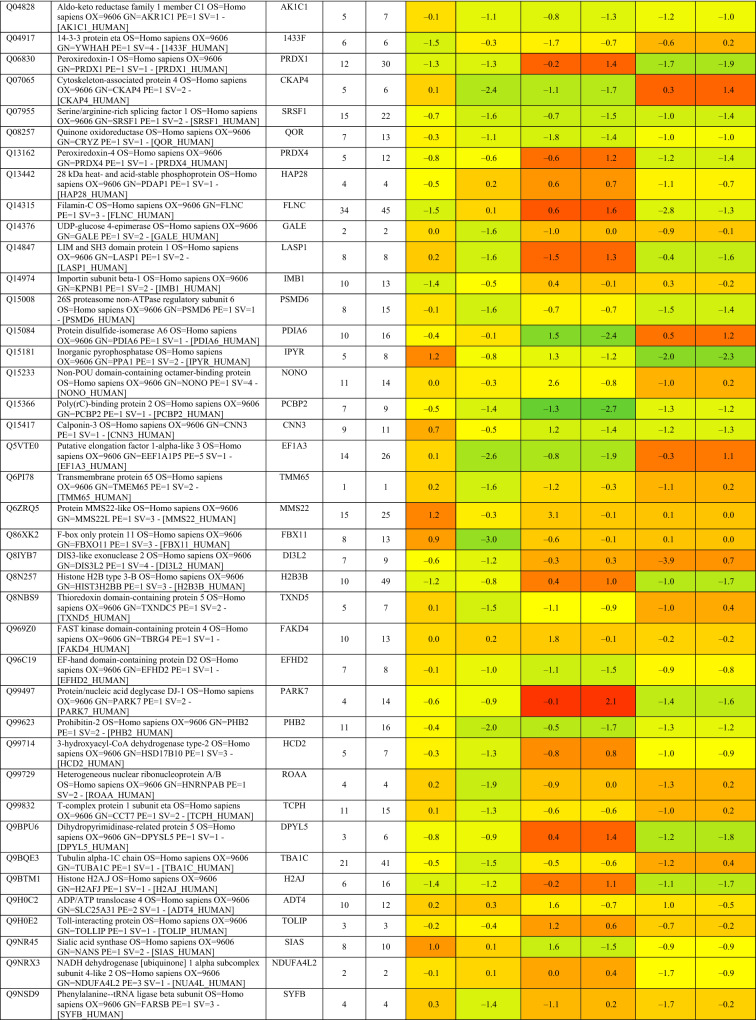

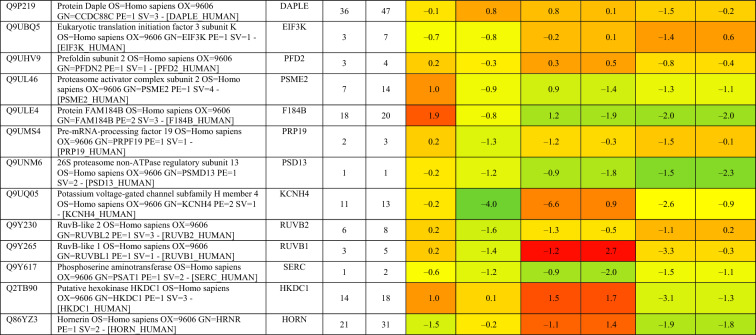
List of proteins expressed in the vero cells post infection, infection and gramicidin S treatment and infection and melittin treatment for 24 and 48 h post infection.

A total of 125 proteins were selected for the Network and pathway analysis involving STRING v11.5. Based on Gene ontological functional enrichment analysis it was found that cellular process (125 proteins), biological regulation (108 proteins) and regulation of biological process (104 proteins) are the most highly associated biological processes; binding (113 proteins), proteins binding (87 proteins) and heterocyclic compound binding (84 proteins) are the most associated molecular functions and cellular anatomical entity (129 proteins), intracellular (125 proteins) and organelle (122 proteins) are the most occurred cellular components. Carbon metabolism (Blue color nodes), pentose phosphate pathway (Blue color nodes) and mRNA processing (Red color nodes) were most prominent local network cluster associated with STRING analysis (Fig. 5b). G6PD, PGK1, ALDOA, ALDOC, TKT, TALDO, LDHB, LDHA, PGAM1 and PGAM2 were the proteins which were found to be associated with carbon metabolism and pentose phosphate pathway analysis. Similarly TRA2B, RBMX, HNRPM, PTBP1, HNRPK, SRSF1, HNRPU, DDX5 and HNRPC proteins were found to be associated with mRNA processing pathway.

### Interaction of gramicidin S and melittin with RBD domain of spike protein: In silico analysis

**Figure 6:**
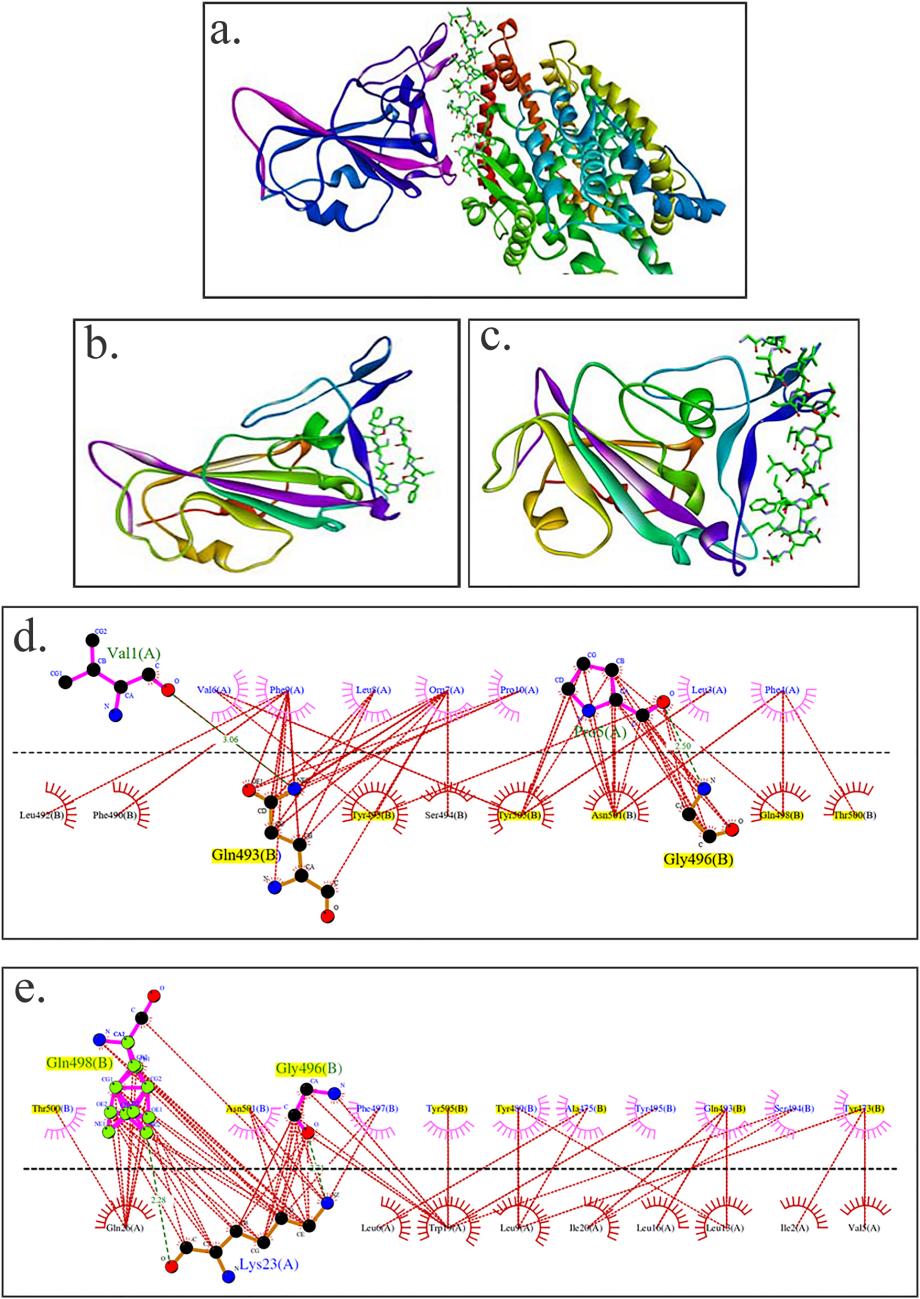
Structures of RBD of spike proteins and peptides and RBD binding region of ACE 2. (**a**) RBD and ACE, (**b**) RBD and gramicidin S, (**c**) RBD and melittin. The ACE2 binding region in RBD is colored violet. In ACE2, gramicidin S and melittin, the peptide regions binding to RBD are represented as sticks. LigPlots of interaction between ACE2 binding domain of the spike protein and (**d**) gramicidin S and (**e**) melittin. The residues of the RBD involved in binding to ACE2 are in yellow. The structures in panel (**a**–**c**) were generated using Discovery Studio v19.1.0.18287. The figures in panel (**d**, **e**) were generated using LigPlot.

Both gramicidin S and melittin are membrane-active peptides and exert their antimicrobial activity by interacting with membrane components^13,14^. Both the peptides may exert their antiviral activity by targeting multiple regions of the virus. The ability of the peptides to bind to the receptor binding domain (RBD) of the SARS-CoV-2 spike protein was examined by molecular docking using web version of the program ZDOCK^27^. The structures shown in Fig. 6 indicate that both gramicidin S and melittin can bind to the RBD binding domain. Both the structures were in the top ten predictions in the ZDOCK output. Panel A shows the crystal structure of RBD-ACE2 complex. The interface between RBD and ACE are shown in violet color and stick representation respectively. The models of gramicidin S and melittin are shown in panels B and C respectively. The LigPlot^28^ of the interacting amino acids are shown in panels D for gramicidin S and E for melittin. The residues highlighted in yellow are involved in RBD binding to ACE2^10^. The modeling study indicates that both the peptides can bind to RBD although their sequences are considerably different from the RBD binding region of ACE2. Both the peptides did not dock to the N-terminal region of ACE2 that binds to RBD region of the spike protein in the top 10 predictions (data not shown).

## Discussion

During the past two decades, the world had witnessed infection by three highly pathogenic human corona viruses namely, SARS-Co-V, MERS, SARS-CoV-2^29,30^. They belong to the group of β-coronavirus and have the ability to cross animal-human barriers and cause serious illness in humans. The timely development of specific antivirals is of utmost importance.The development of vaccines at “warp speed” has led to decrease in mortality and serious illness caused by SARS-CoV-2^3^. However, it is still not well established that whether vaccines are equally effective against the several variants that are emerging, such as the omicron variant, or the time frame when immunity will be present. To-date, there is no specific drug for SARS-CoV-2. Hence, there is clearly an urgent need to develop therapeutic molecules that would effectively neutralize the virus rather than repurposing drugs used to treat other viral infections. In case of HIV, drugs specific to the virus has been effective to treat the disease although there is still no effective vaccine. Likewise, malaria can be treated with specific drugs rather than vaccines.

However, development of specific therapeutic antiviral drugs for clinical use in a short span is extremely challenging. Repurposing of drugs already known for their therapeutic effects have been extensively screened and tested for inhibition of SARS-CoV-2^1,6^. While many repurposed drugs have shown excellent anti SARS-CoV-2 activity in vitro, they have shown very little success when used clinically. We have shown that host defence peptide such as β-defensins may have a role in infection by SARS-CoV-2 as they are down regulated in COVID-19 patients^31^.

Bee venom has immunosuppressive activity and is generally used in contemporary medicine to treat Multiple Sclerosis, Parkinson's disease, and arthritis. Bee venom activates foxP3-expressing cells, CD25 and CD4 + T cells, and thus modulates the IgE antibody ratio, resulting in a variety of allergic reactions to antigens^32^. This immunosuppressive activity was observed in Wuhan beekeepers against COVID-19^32^. Melittin is the main component of bee venom, and it is active against both enveloped and non-enveloped viruses by activating the Toll-like receptors (TLRs) pathway, which reduces inflammatory cytokines like nuclear factor-kappa B (NF-kB), extracellular signal-regulated kinases (ERK1/2), and protein kinase Akt^32^. Melittin exhibited antiviral activity against several viruses in vitro^18^. Significant antiviral activity of the sitagliptin and melittin-nanoconjugates complex (IC50 value-8.439 µM) was observed against SARS-CoV-2 in vitro^19^. Binding of sitagliptin and melittin nano conjugates to the active site of SARS-CoV-2 3CLpro (a protease) was also observed through molecular docking^19^.

Gramicidin S has potent antibacterial and fungicidal activity^13^. Molecular docking revealed that gramicidin S has a binding affinity of 11.4 kcal/mol to the SARS-CoV-2 spike glycoprotein and SARS-CoV-2 papain like protease, implying that gramicidin S could be an effective drug against the SARS-CoV-2 virus^33^. SARS-CoV-2 is an enveloped virus, with the viral membrane essential for its integrity and function^1,10^. We reasoned that membrane-active peptides would disrupt the viral membrane and render the virus ineffective. We have investigated the anti-SARS-CoV-2 activity of two well studied membrane-active antibacterial peptides gramicidin S and melittin.

Our present in vitro studies using gramicidin S and melittin showed EC_50_ value of 1.571 µg for gramicidin S and 0.656 µg for melittin (Fig. 1). The results were comparable with remdesivir which we used as the assay control in our study (Fig. 5). The immunofluorescence studies are in agreement with our RT-qPCR data showing the decrease in the viral load in the gramicidin S and melittin treated experimental groups compared to peptide non treated group (Fig. 4). Molecular docking studies suggest that both the peptides have structural features that would favor binding to RBD of the spike protein (Fig. 6). ZDOCK used in the present study, has been used extensively to study protein–protein/peptide interactions^34^. However, our prediction that melittin and gramicidin S have structural features that favor interaction with RBD needs experimental validation. In fact, membrane-active antibacterial peptides have multiple targets in bacteria. It is conceivable that gramicidin S and melittin also have multiple targets on the virus thereby acting as an effective antiviral agent. Both gramicidin S and melittin have hemolytic activity at concentrations higher than the EC50 values reported in this study. Hemolytic activity can be attenuated or eliminated by engineering the peptide for specific antiviral activity. This approach has been successfully used in generating gramicidin S and melittin analogs with only antibacterial activity without any hemolytic activity^35,36^. In order to prevent off-target toxic effects of melittin, formulations with polymeric nanoparticles are being explored^37^. Formulations for use of peptides as therapeutics is a challenge but there are considerable efforts towards this goal^12^.

Proteomics studies indicate that metabolic change caused by SARS-CoV-2 pathogenesis result in long-term metabolic disorders in COVID-19 patients, and this varies according to pathogen severity. Carbon sources, specifically glycolysis and glutamino lysis pathways, have been found to play critical roles in SARS-CoV-2 viral replication and production^38^. Non-oxidative pentose phosphate pathways (PPP) are also involved in viral replication; Transketolase is a key mediator enzyme of PPP involved in ribonucleotide production. Benfo-oxythiamine, a TKT inhibitor, acted against SARS-CoV-2 infection and inhibited the viral replication^39^. Our findings from proteomic pathway analysis showed that several proteins are strongly associated with carbon metabolism and non-oxidative PPP. Specifically, LDHA, LDHB, ALDOA, TALDO, PGK1, and PGAM2 proteins were found to be up regulated in early viral replication, i.e. at 24 h viral induced cell control, and vice versa in gramicidin-treated cells. TKT was found to be up regulated after 48 h of viral induced cell control, but it was substantially down regulated by melittin treatment. It suggests that gramicidin and melittin may function as viral inhibitors by suppressing intercellular metabolic regulators.

Another prominent pathway, mRNA processing, was identified during the local network pathway analysis. According to a recent study on SARS-CoV-2 RNA host protein interaction, the majority of virus which induced host RNA binding proteins prevents the virus induced cell death. Several mRNA binding proteins, including Heterogeneous nuclear ribonucleoproteins (HNRNPs), dead box RNA helicases (DDX), and NONO, were activated during the innate immune response to SARS-CoV-2 infection^40^. In our study, NONO, DDX5, RBMX, and HNRPM proteins were found to be up regulated with more than 1 log fold change in melittin-treated SARS-CoV-2 infected cells. This finding suggests that melittin may have antiviral activity during the early stages of viral infection by activating host RNA binding proteins.

The antimicrobial activity of gramicidin S and melittin have been well characterized. Our results strongly argue for development of peptides, gramicidin S and melittin, as potent therapeutic candidates to treat SARS-CoV-2 and possibly other influenza like viruses which are also enveloped viruses, for which there are no effective vaccines. Localized delivery at the site of infection in the nasopharyngeal region by appropriate formulations would avoid cytotoxicity due to systemic delivery.

In conclusion, our study indicates the potential of antibacterial peptides such as gramicidin S and melittin for development as therapeutic molecules to treat COVID19. These peptides have broad-spectrum antibacterial activity and resistance does not develop against them. It is likely that variants of SARS-CoV-2 which may escape immune surveillance, may be susceptible to membrane-active peptides such as gramicidin S and melittin. With tremendous advances in the formulation of drugs to minimize side-effects, it should be able to administer gramicidin S and melittin by appropriate formulations to avoid any non-specific cytolytic effects.

## Materials and methods

### Peptides

Gramicidin S and melittin were procured from commercial sources (Gramicidin S: 368108, Calbiochem CA, USA Melittin: M4171 from Sigma Chemical Co, USA). They were characterized by HPLC and mass spectrometry and found to be > 95% pure.

### Cell viability using MTT assay

The Vero cells were plated in 96 well culture plate and incubated at 37 °C with 5% CO_2_. After attaining 90–95% cell confluency, different concentrations of gramicidin S and melittin (0.5, 0.7, 3, 5 µg for both) were added to the cells to check the effect of the peptides on the cells for 24 h. After 24 h, 100 µl (50 µg) of MTT substrate was added to the cells and the plate was continued to incubate for 3 h at 37 °C with 5% CO_2_. Later the formazan crystals formed were dissolved in 100 µl of DMSO and the absorbance was measured at 570 nm in Multimode Micro plate reader (Synergy HIM).

### RT-qPCR assay

The effect of gramicidin S and melittin was tested against the SARS-CoV-2 with different concentrations. Remdesivir was run as an assay control. The titers for the virus were adjusted such that there was only viral replication and no cytolysis. Briefly, the virus (MOI 0.1) was pre-incubated with different concentrations of gramicidin S and melittin (0.1–10 µg) for an hour at 37 °C. After the incubation, virus inoculum containing gramicidin S and melittin was added to the Vero cells in duplicates (50 µl/well). Remdesivir (1 µM) was added to the Vero cells without pre-incubation as in the case of peptides. All the experimental groups were left for infection for 3 h while maintaining at 37 °C with 5% CO_2_. Post-infection (PI), media containing viral inoculum and the gramicidin S and melittin was removed and replaced with 200 µl of fresh DMEM media containing 10% FBS and the experimental groups were maintained for varying time points in an incubator maintained at 37 °C with 5% CO_2_. Post-incubation, cell supernatants from the experimental groups were collected and spun for 10 min at 6000 g to remove debris and the supernatant was transferred to fresh collection tubes and later were processed to isolate viral RNA. RNA was extracted from 200 μL aliquots of sample supernatant using the MagMAX™ Viral/Pathogen Extraction Kit (Applied Biosystems, Thermofisher). Briefly, the viral supernatants from the test groups were added into the deep well plate (KingFisher^TM^Thermo Scientific) along with a lysis buffer containing the following components—260 μL, MagMAX™ Viral/Pathogen Binding Solution; 10 μL, MVP-II Binding Beads; 5 μL, MagMAX^TM^Viral/Pathogen Proteinase-K, for 200 μL of sample. (Extraction was performed using KingFisher Flex (version 1.01, Thermo Scientific) according to manufactures instructions). The eluted RNA was immediately stored in – 80 °C until further use.

The detection of SARS-CoV-2 was done using COVID-19 RT-PCR Detection Kit (Fosun 2019-nCoV qPCR, Shanghai Fosun Long March Medical Science Co. Ltd.) according to the manufacturer’s instructions. The kit detects Envelope gene (E; ROX labelled), Nucleocapsid gene (N-JOE labelled) and Open Reading Frame1ab (ORF1ab, FAM labelled) specific to SARS-CoV-2 for detection and amplification of the cDNA. SARS-CoV-2 cDNA (Ct ~ 28) was used as a positive control. The log viral particles and a semi–log graph was plotted through the linear regression equation obtained using the RNA extracted from the known viral particles by RT-qPCR, using N- gene specific to SARS CoV-2 virus.

### Immunocytochemistry

The Vero cells were seeded in 6-well plate with the sterile glass cover slips. Cells at 90–95% confluency were considered for SARS-CoV-2 infection. Briefly, gramicidin S and melittin, were pre-incubated with SARS-CoV-2 virus for 1 h at 37 °C with 1.5 µg/100 µl and 3 µg/100 µl respectively. Later, the viral inoculum incubated with peptides were used to infect Vero cells on the glass coverslips. After 3 h of infection, the viral inoculum containing the peptides was replaced with fresh media with 10% FBS until 12 and 24 h. Parallel controls were maintained without the drug treatment. After 12 and 24 h, the treated and untreated cells were fixed with 4% paraformaldehyde and processed further for immunocytochemistry. The fixed samples were washed thrice with PBS and the cells were permeabilized using 0.3% Triton X-100 (Sigma, cat. no.: X100; Lot no.: 056K0045) in PBS for 15 min at room temperature (RT). Then the cells were washed with PBS, thrice for 5 min each at RT. The cells were incubated with the 3% Bovine Serum Albumin (BSA) (Sigma) in PBS, for 1 h at RT to block the nonspecific antibody binding. Later the experimental groups were incubated with the anti-sera for RBD of SARS-CoV-2 (1:200) prepared in 1% BSA made in PBS (anti-sera against SARS-CoV-2 was raised in rabbits and validated using ELISA at CCMB) overnight at 4 °C. After incubation the cells were washed with PBST, thrice at RT for 10 min each. Later the cells were incubated with the secondary anti-Rabbit IgG antibody conjugated with Alexa Fluor 488 (Life Technologies, Cat. no.: A11008; Lot no.: 1735088) at the dilution of 1:200 in 1% BSA made in PBS. Rhodamine Phalloidin (Life Technologies, Cat. no.: R415; Lot no.: 1738179) was used to label F-actin. The cells were incubated with secondary antibody and Rhodamine phalloidin mix for 1 h at room temperature. After incubation the cells were washed with PBST, thrice at RT for 10 min each. Then, the cover slips containing the cells were mounted over the pre-cleaned slides using Vectashield mounting medium containing DAPI (for nuclear staining) (Vector Laboratories, Cat. no.: H-1200, Lot no.: ZC1216). Images were obtained using confocal microscope FV3000 with software version 2.4.1.198 (Olympus Life Sciences Solutions) in Light Scanning Microscopy (LSM) mode.

### Proteomic analysis

Total protein was extracted from the control Vero cells, Vero cells infected with SARS-CoV-2 and Vero cells infected with SARS-CoV-2 and treated with gramicidin S and melittin separately. The cells were collected at 24 and 48 h independently. The samples were centrifuged and pellet was dissolved in protein solubilisation buffer^41,42^ and sonicated for 10 min at BSL3 lab facility. The protein samples were further centrifuged for 30 min at 14,000 RPM to remove the cell debris. Quantification of the pooled protein samples were performed using Amido Black method against BSA standard. A 200 µg of total protein from all the experimental groups were electrophoresed in 10% SDS-PAGE, Commassie R250 stained, destained and gel excised in to four fractions based on molecular weight. In-gel trypsin digestion and iTRAQ labeling and purification were performed as described earlier^42–44^. iTRAQ label was labelled as 114—Control; 115—Infection; 116—Infected cells treated with Melittin and 117—Infected cells treated with gramicidin S. All labelled peptides were pooled and purified by running through C18 column. Peptides were reconstituted in 5% acetonitrile (ACN) & 0.2% formic acid and then subjected to the Liquid Chromatography Mass Spectrometry (LCMS/MSMS) analysis in OrbitrapVelos Nano analyzer (Q-Exactive HF). The proteomic data obtained from the mass spectrometer were analysed against human proteome and SARS-CoV-2 proteome data. All the obtained proteome data were tabulated and differential expression in SARS-CoV-2 proteins were estimated against the control negative samples. The obtained proteome data was analysed for its heat map expression profile using heatmapper software^45^ (www.heatmapper.ca). Network and pathway analysis of the associated proteins were performed using STRING v11.5^46^ (https://string-db.org).

### Molecular docking

The receptor binding domain (RBD) of the SARS-CoV-2 spike protein was obtained by editing the crystal structure of the C-terminal domain of the SARS-CoV-2 spike protein in complex with human ACE2 (PDB id: 6zlg)^47^. The ID of the structure used for gramicidin S monomer is CCDC 626343. Monomeric melittin structure was obtained by editing the crystal structure of tetrameric melittin (PDB id: 2mlt). The structures were generated using Discovery Studio v19.1.0.18287 (2019). Interactions between amino acids were visualized using LigPlot^28^.

### Statistical analysis

All the experiments were performed in duplicates with technical replicates (n = 6). The data analysis and graphs were generated using GraphPad Prism (*Ver* 8.4.2). All the values were represented as mean ± SD.

### Ethics approval

The Anti-SARS CoV-2 study was approved from Institutional Bio-safety Committee of CSIR-Centre for Cellular and Molecular Biology, Hyderabad, India.
